# Acoustic Signatures of Hive: Detecting Queen Bee Absence Through Machine Learning of Short Audio Segments

**DOI:** 10.3390/insects17060547

**Published:** 2026-05-25

**Authors:** Pablo Ormeño-Arriagada, Cristopher Jiménez, Ramón Arias Gilart, Daniel Ramírez, Karen Yañez

**Affiliations:** 1 Ingeniería Civil Informática, Facultad de Ingeniería, Negocios y Ciencias AgroAmbientales, Universidad de Viña del Mar, Viña del Mar 2520000, Chile; pablo.ormeno@uvm.cl; 2 Centro de Biotecnología Dr. Daniel Alkalay Lowitt, Universidad Técnica Federico Santa Maria, Avenida España 1680, Valparaíso 2390123, Chile; cristopher.jimenez@usm.cl (C.J.); ramon.arias@usm.cl (R.A.G.); 3 Departamento de Ingenieria Química y Ambiental, Universidad Técnica Federico Santa María, Avenida España 1680, Valparaíso 2390123, Chile; daniel.ramirez@usm.cl

**Keywords:** acoustic-based, classification, machine learning, deep learning, MFCC, precision apiculture

## Abstract

Honeybee population decline poses a serious threat to global biodiversity and agricultural productivity, underscoring the need for continuous and non-invasive hive monitoring solutions. In particular, early detection of queen absence is critical for maintaining colony viability. This study investigates the effectiveness of machine learning and deep learning models for acoustic-based queen-presence detection using short-duration hive audio recordings. Audio data collected from multiple sources were processed to extract spectrogram, Mel-spectrogram, and Mel-frequency cepstral coefficient features, which were evaluated using classical ML classifiers and convolutional neural networks. Experimental results indicate that MFCC-based representations consistently outperform spectrogram-based features across segment lengths, achieving higher accuracy and greater stability. The best performance was obtained with Mel features using convolutional neural networks for short segments and gradient-boosted models for longer windows. These findings demonstrate that brief acoustic segments are sufficient for reliable classification, supporting real-time monitoring under realistic urban recording conditions with moderate environmental noise. The proposed approach offers a scalable and low-cost framework for precision beekeeping and contributes to sustainable beekeeping through early, automated anomaly detection. The proposed framework supports real-time, low-cost deployment scenarios, enabling scalable precision apiculture solutions.

## Simple Summary

Honeybee colonies are essential for pollination and food production, yet their populations are declining worldwide. One of the key factors affecting colony survival is the loss of the queen, which can quickly lead to colony collapse if not detected in time. Traditional inspection methods are time-consuming, invasive, and require expert knowledge. In this study, we explore the use of sound recordings from beehives combined with machine learning techniques to automatically detect the presence or absence of the queen. By analyzing short audio segments, we found that specific sound features can reliably indicate queen status, even under noisy field conditions. Our results show that these methods can provide accurate and stable detection using low-cost and scalable technologies. This approach offers a practical tool for beekeepers, enabling continuous and non-invasive monitoring of hive health, and supporting more sustainable and efficient beekeeping practices.

## 1. Introduction

Pollination is a fundamental process for biodiversity conservation and occurs through abiotic mechanisms, such as wind or water, and biotic interactions involving animals. According to [1], approximately 90% of flowering plant species rely on animal pollination, with honeybees playing a dominant role and contributing to 87% of global food production. Furthermore, other authors concluded that more than 100 important crops depend on pollination by honeybees [2]. Beyond agriculture, their relevance extends to beekeeping and textile-related ecosystems. Honeybees are also recognised as keystone species in terrestrial environments, promoting ecological stability through cross-pollination and increased genetic diversity [3]. Their ongoing decline poses serious risks to food security, ecosystem services, and biodiversity resilience. Therefore, scientifically grounded and technology-driven analyses of bee colony dynamics are essential to sustain environmental balance and global agricultural productivity [4].

Monitoring systems are essential for understanding hive dynamics by continuously measuring parameters such as weight, temperature, humidity, vibration, and sound [5,6]. In particular, weight data provide insights into resource accumulation and consumption [7], while acoustic and vibrational analyses enable the detection of swarming events [8,9]. Additionally, temperature and humidity measurements reveal brood distribution and thermoregulation processes [10,11]. In parallel, recent advances in machine and deep learning have enabled accurate modelling of multimodal sensor data, supporting classification and prediction tasks in apiculture. For example, deep learning has been applied to beehive monitoring [12], pollution assessment via acoustics [13,14], and hive weight estimation without scales through sensor fusion [15]. Overall, AI-based acoustic analysis allows continuous, real-time detection of anomalies and anticipates key colony events, improving beehive status and productivity [16,17,18].

Technological advances related to the monitoring of apiaries are remarkable today. However, global honeybee populations are declining annually due to multiple stressors, including diseases [19], climate change [20], and pollution [13]. In this context, continuous hive monitoring is critical for early anomaly detection [21], particularly for identifying queen bee absence [22], which is essential for colony viability. The loss of the queen can lead to the emergence of laying worker colonies, resulting in severe disruptions to reproduction and overall colony health [22]. Despite advances in sensor technologies and data analytics, integrated real-time systems capable of reliably detecting queen absence from short-duration acoustic patterns under natural environmental conditions remain limited [4,18].

To address this challenge, Machine Learning (ML) and Deep Learning (DL) approaches have been applied to audio data collected from hives with and without queen bees. The analysis leverages acoustic representations such as logarithmic spectrograms, Mel spectrograms, and Mel-frequency cepstral coefficients (MFCCs), extracted from short audio segments ranging from 1 to 3 s. This data-driven methodology enhances the detection of colony irregularities, particularly those associated with queen absence, thereby supporting early intervention strategies. Ultimately, such approaches contribute to improving the condition of the hive, its resilience, and the long-term sustainability of honeybee populations on a global scale [18].

This study makes several methodological and practical contributions to the field of bioacoustic monitoring and intelligent apiculture:

- Multi-source dataset integration. We integrate heterogeneous audio recordings from three distinct sources—Hiveeyes, LongHive, and the USM Bee Lab—representing different recording conditions, geographical locations, and bee subspecies. This cross-source design enhances the ecological validity and generalizability of the models, addressing a major limitation of prior studies that relied on single, controlled datasets.
- Systematic comparison of ML and DL approaches. We conduct a comprehensive evaluation of both classical ML algorithms and DL architectures under a unified preprocessing pipeline. This comparison clarifies the relative strengths of shallow versus hierarchical models for acoustic pattern recognition in noisy field data.
- Feature study across multiple temporal resolutions. The research examines three complementary acoustic representations—spectrogram, Mel-spectrogram, and MFCCs, applied to short audio segments of 1, 2, and 3 s. This design provides insights into the trade-off between temporal context and computational efficiency, revealing how perceptually motivated features (Mel, MFCC) perform under constrained time windows.
- Field validation under real environmental noise. Unlike previous laboratory-based experiments, we validate the models using in situ recordings from active hives located in urban environments. These datasets include realistic background noise (vehicular traffic, ambient sounds), demonstrating the robustness and applicability of the proposed system under operational field conditions.

Overall, this research advances bioacoustic intelligence by establishing a reproducible, low-cost, and scalable framework for real-time queen bee detection. Through the integration of multisource datasets, diverse acoustic features, and cross-model evaluation, the study contributes to the development of smart beekeeping systems capable of enhancing colony management, early anomaly detection, and pollinator sustainability.

Despite advances in hive monitoring and the growing use of machine learning and deep learning in apiculture, key gaps persist. First, most systems prioritise long-term or coarse-grained indicators, limiting the capture of short-term behavioural signals essential for early anomaly detection. In particular, although acoustic analysis shows strong potential for identifying swarming, disease, or environmental stress, short-duration audio segments under real-world conditions remain underexplored. Second, many AI-based approaches depend on isolated modalities or complex sensor fusion, increasing cost and deployment barriers. Consequently, lightweight, real-time solutions capable of inferring critical colony states—such as queen presence or absence—from minimal acoustic data are scarce. Finally, despite its decisive role in colony viability and reproduction, queen absence has received limited attention from an acoustic, data-driven perspective, motivating the need for novel, robust detection frameworks. Unlike prior studies relying on long-duration recordings or controlled environments, this work demonstrates that queen presence can be reliably inferred from short-duration acoustic segments (1–3 s) under real-world field conditions using a unified multisource dataset. Existing approaches rely on long-duration recordings or controlled environments, limiting their applicability for real-time, field-based monitoring. The feasibility of detecting queen presence from short-duration acoustic segments under realistic conditions remains insufficiently explored.

The remainder of this paper is organised as follows. Section 2 reviews related work on honeybee monitoring systems, with a focus on acoustic analysis and machine learning approaches for a good condition of the beehive. Section 3 describes the proposed methodology, including data acquisition, acoustic feature extraction, and the machine learning and deep learning models used for queen absence detection. Section 4 presents the experimental results and performance evaluation of the proposed approach. Section 5 discusses the findings in relation to existing literature, highlighting practical implications and limitations. Finally, Section 6 concludes the paper and outlines directions for future research.

## 2. Related Work

Acoustic monitoring has emerged as a non-invasive proxy for hive state, with recent reviews synthesising feature engineering (MFCCs, Mel-spectrograms), model families, and deployment constraints under realistic noise and hardware limits [16]. Beyond reviews, efforts in dataset curation and harmonisation aim to improve cross-hive generalisation; for example, BeeTogether standardises Queen/NoQueen labels across sources and exposes extrapolation challenges [23]. Methodologically, Convolutional Neural Networks (CNNs) trained on compact time–frequency representations have advanced queen-status classification from short clips [24], while comparative studies benchmark CNNs against classical ML using engineered spectral features [25]. More recently, compact CNNs address real-time, embedded constraints [26], and large multimodal urban datasets enable analyses of robustness and seasonality in bioacoustic inference.

### 2.1. Acoustic Monitoring of Hives

Recent studies have demonstrated that acoustic signals emitted by honeybee colonies provide reliable indicators of good hive condition, as hives generate characteristic sounds linked to collective activities, including queen presence [27]. More broadly, these acoustic signatures function as non-invasive bioindicators of colony behaviour, enabling the early detection of stress, disease, and environmental disturbances within the hive ecosystem [28]. Consequently, sound-based monitoring offers a continuous and unobtrusive means of assessing colony state, complementing traditional inspection methods and supporting timely management interventions.

### 2.2. Machine Learning on Hive Audio

Machine learning techniques have been applied to the analysis of beehive audio data to automatically identify patterns and anomalies in acoustic signals. In this context, learning-based algorithms enable the recognition of sound signatures associated with different hive states, supporting the early detection of events such as queen absence or behavioural changes within the colony [27]. Moreover, ML provides scalable and objective frameworks for real-time anomaly detection, allowing continuous monitoring of colony stress and stability without the need for manual inspection [28,29].

### 2.3. Deep Learning for Audio/Spectrograms

Several studies have investigated automated queen detection using ML and DL techniques. For instance,  [24] proposed a deep CNN that predicts queen presence from audio signals by leveraging summarised spectrogram representations, achieving superior performance compared to classical machine learning approaches. In a comprehensive review, ref. [16] surveyed automated beehive acoustic analysis methods, highlighting the use of MFCCs for tasks such as bee detection, queen absence identification, and swarming recognition. Complementarily, ref. [30] introduced two vision-based methods for queen detection in beehive frames, combining hand-crafted features with traditional machine learning in one approach and a LeNet-5-inspired deep neural network in the other.

### 2.4. Multimodal Systems (Sound and Environmental Sensors)

Frequency-based analysis of hive acoustics, including spectrogram representations, enables the extraction of informative patterns linked to specific colony conditions. In particular, variations across frequency bands can be correlated with behavioural states and anomalies within the hive. Moreover, acoustic analysis has increasingly been combined with other sensory modalities, such as temperature and humidity, to achieve a more comprehensive understanding of hive dynamics. As a result, multimodal approaches have gained prominence for improving the accuracy and robustness of hive status monitoring systems. For example, [31] proposed a non-invasive monitoring framework that detects queen absence by analysing beehive sound signals using a classifier. This system integrates dedicated hardware, signal processing algorithms, and ML techniques, demonstrating the effectiveness of acoustics-driven, sensor-assisted solutions for automated colony assessment.

### 2.5. Synthesis and Gap

In [22,32], acoustic data from beehive colonies were analysed across three classes—Bee, NoBee, and NoQueen—using a diverse set of spectral and cepstral features, including spectral centroid, zero-crossing rate, chromagram, constant Q-transform, and MFCCs. To enhance discrimination, hybrid feature-selection techniques such as principal component analysis (PCA), chi-square, and singular value decomposition (SVD) were applied. As a result, ensemble and instance-based classifiers, particularly Random Forest and K-Nearest Neighbors (KNN), achieved the best performance. In parallel, ref. [33] provided a comprehensive overview of computer vision and machine learning approaches for bee monitoring, highlighting the growing role of data-driven methods in automated colony assessment. Despite advances in acoustic monitoring and machine learning, existing studies predominantly rely on long-duration recordings, controlled environments, or single-source datasets. Consequently, the reliability of short-duration acoustic inference under real-world conditions remains unclear.

## 3. Materials and Methods

The experimental design was structured to evaluate whether short-duration acoustic segments (1–3 s) contain sufficient information for reliable queen-presence detection under realistic field conditions. To this end, multiple feature representations and model families were evaluated across segment lengths. Audio recordings were processed following established bioacoustic analysis pipelines, ensuring reproducible feature extraction and segmentation. Consistent with recent beehive acoustic studies, recordings were resampled to 44.1 kHz and normalised to mitigate amplitude variance before time–frequency transformation using Short-Time Fourier Transform (STFT) and Mel filterbanks [34]. Each signal was partitioned into non-overlapping 1–3 s segments to balance temporal resolution with computational efficiency, a design shown to capture colony-level behavioural cues while maintaining robustness to environmental noise [25]. From each segment, spectrogram, Mel-spectrogram, and MFCCs were derived and standardised. These features were used to train and evaluate both classical and deep learning classifiers under identical preprocessing conditions.

### 3.1. Datasets

The training audio data were collected from two complementary sources. First, the Hiveeyes project [35] provided 20 recordings from 11 urban hives in Berlin, located near a low-traffic street and hosting Apis mellifera carnica or carnica–Buckfast hybrids. Audio durations ranged from 32 to 90 s, with five recordings corresponding to queenless hives. Files were stored in mp3 format at a sampling rate of 44.1 kHz, capturing frequencies up to 22.05 kHz. Second, the LongHive project [36] contributed 35 recordings from the “To bee or not to bee” dataset [37], collected from two hives, each lasting approximately 9:50 min and stored in wav format. Overall, the combined dataset comprises approximately 6 h of hive audio, with most Hiveeyes samples sourced from https://community.hiveeyes.org/t/sound-samples-and-basic-analysis-hive-with-queen-vs-queenless/399 (accessed on 10 March 2026).

The testing dataset was collected from two hives, Hive A and Hive B, located at the USM Bee Lab apiary of the Universidad Técnica Federico Santa María in Valparaíso, Chile, approximately 100–200 m from a high-traffic avenue. Consequently, recordings contain moderate environmental noise and reflect realistic operating conditions. Audio data were acquired between 3:00 p.m. and 8:00 p.m. on 2 December and 9 December 2022, both sunny days. Regarding hive status, Hive A contained a queen throughout the observation period (25 November to 9 December 2022). In contrast, Hive B was queenless prior to 2 December, although queen larvae were present, and a new queen emerged by 9 December. Thus, the dataset captures dynamic queen-state transitions under natural conditions. The evaluation dataset is imbalanced, comprising approximately 500 queen-absent (SA) and 250 queen-present (CA) samples across segment durations. Data were acquired in situ from active urban beehives, incorporating traffic, wind, and heterogeneous recording conditions. Unlike controlled studies, recordings include non-apicultural noise, prioritizing real-world generalisation. Urban noise introduces low-frequency and broadband components that affect linear spectrogram models. Perceptually scaled representations (Mel spectrograms, MFCCs) improve robustness by emphasizing colony-relevant patterns and compressing broadband noise via Mel scaling and DCT [38]. Short temporal windows (1–3 s) further reduce persistent noise effects. Models were trained on multi-source data with varying acoustic conditions, maintaining stable performance. While isolating specific noise sources requires controlled experiments, this study focuses on realistic deployment scenarios without curated noise separation.

**Figure 1:**
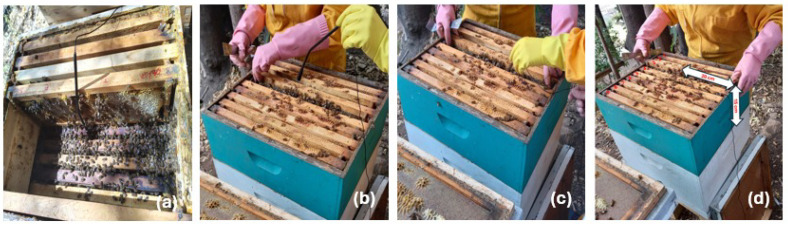
Test with different position between frames (**a**); hive without queen (**b**); Installation of the microphone between frames most representative of the worker population (**c**); and distance of microphone between brood chamber and honey super (**d**).

Obtaining audio was achieved by installing a microphone inside each hive mentioned in the previous point. The microphones used were Trust brand fine design desktop microphones, specifically, an omnidirectional condenser microphone with a signal-to-noise ratio of 58 dB, frequency response of 100 Hz–12,000 Hz, impedance of 2200 Ohm, sensitivity of −45 dB, pressure level noise level of 115 dB, cable connection, connector type 3.5 mm, cable length 1.8 m, total weight 47 g, without noise reduction, with reference number 21,674. The microphone was installed between frames 4 and 5 or 5 and 6 (depending on the location of the core of each hive) at a distance of approximately 15 cm, taking care to capture the sound of the hive between the brood chamber and the honeycomb, as shown in Figure 1.

The sampling rate used was 44,100 samples per second; to record the hives a Galaxy A31 phone and a LENOVO ideapad 330S laptop with Microsoft Windows 10 Home Single Language operating system Intel(R) Core(TM) i7-8550U processor were used. The audio samples collected on those dates were as follows:

- 2 December 2022: Twenty recordings were collected. One of them corresponds to Hive A (recorded on the phone) and the rest correspond to Hive B (recorded on the laptop and phone). The minimum audio duration is 8 [s] and the maximum duration is 1:21 min.The extension of the audio files obtained from Hive A is .m4a and the extension of the audio files obtained from Hive B is .mp3 and .m4a.
- 9 December 2022: Fifty six recordings were collected. Twenty one of them correspond to Hive A (recorded on the laptop) and the rest correspond to Hive B (recorded on the phone). The minimum audio duration is 26 [s] and the maximum duration is 600 [s]. The extension of the audio files obtained from Hive A is .mp3 and the extension of the audio files obtained from Hive B is .m4a. When combining the audio files obtained from Hive A and Hive B, as mentioned above, there are approximately 5:30 [h] of recordings of bee buzzing.

#### Labeling

The original file names were replaced using a structured naming convention to reflect hive conditions. The status field specifies the colony state: *SA* for queen-absent hives and *CA* for queen-present hives. Subsequently, all recordings were segmented into short audio samples of 1, 2, and 3 s. Finally, samples sharing the same duration were grouped to form independent datasets for model training and evaluation.

### 3.2. Audio Characterisation

To implement the processing pipeline, the audio recordings from the Hiveeyes Community, the LongHive project [36], and the UTFSM apiary [39] were first segmented into fragments of 1, 2, and 3 s using Python (version 3.10) based scripts. Subsequently, samples with identical durations were grouped to form separate training and testing datasets for each temporal resolution. Then, a second Python script was used to extract acoustic features from each dataset. Specifically, three representations were computed for every segment length: Mel spectrograms, 40 MFCCs derived from the amplitude time series, and 40 MFCCs derived from the Mel spectrograms. As a result, three feature sets were obtained per duration, yielding a total of nine datasets used for model training and evaluation. Each feature dataset was systematically divided into two mutually exclusive subsets: a training dataset used for model learning and a testing dataset reserved for performance evaluation (see Table 1). MFCCs were computed using the librosa library to obtain compact and perceptually relevant representations of the audio signals. The extraction process employed a short-time Fourier transform with $n_{\mathrm{fft}}=2048$ and a hop length of 512 samples, enabling an appropriate balance between time and frequency resolution. A total of $n_{\mathrm{mfcc}}=13$ coefficients were retained, as commonly used in bioacoustic and speech analysis tasks, capturing the most informative spectral characteristics of the signal. Cepstral coefficients were derived using a Discrete Cosine Transform (DCT type II), which facilitates decorrelation and compression of spectral information, enhancing robustness for subsequent classification.

**Figure 2:**
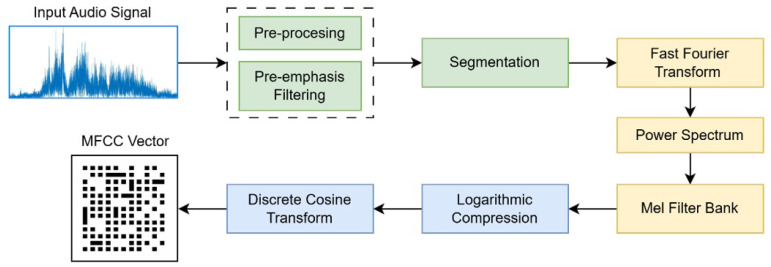
Signal-processing pipeline for Mel-frequency cepstral coefficient (MFCC) extraction, including audio pre-processing, spectrogram computation, Mel-scale transformation, logarithmic compression, and discrete cosine transform.

With successive steps ranging from signal pre-processing to spectral energy computation, a time–frequency representation is obtained in the form of a spectrogram. Subsequently, the application of the Mel scale maps the frequency axis according to human auditory perception, producing a Mel spectrogram. Next, logarithmic compression transforms spectral amplitudes into decibel units, improving perceptual relevance and numerical stability. Finally, the Discrete Cosine Transform (DCT) decorrelates the spectral components, yielding Mel-frequency cepstral coefficients (MFCCs). As a result, MFCCs provide a compact and perceptually grounded representation that has been widely adopted in speech and speaker recognition, music information retrieval, and bioacoustic analysis [42,43]. Figure 2 summarises this signal-processing pipeline from time-domain audio to MFCC extraction and providing a structured overview of the data acquisition, feature extraction, and classification stages.

**Figure 3:**
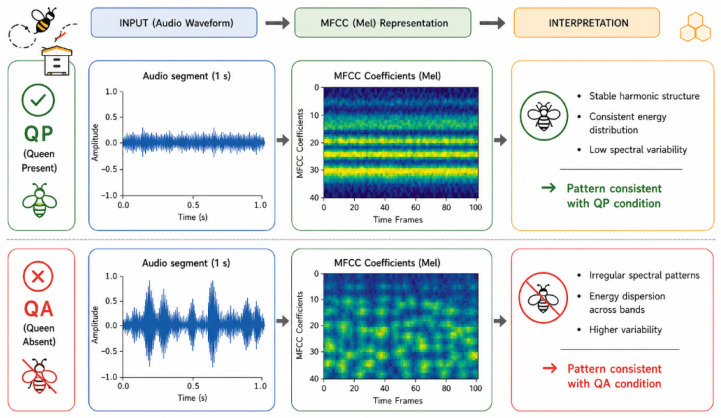
Illustrativeexamples of the acoustic feature extraction process. Each row shows an input audio segment (**left**), its MFCC (Mel) representation (**center**), and a qualitative interpretation (**right**). QP samples exhibit more structured spectral patterns, whereas QA samples show increased irregularity and dispersion. These examples are provided for visual interpretation only and do not represent the full variability of the dataset.

To provide an intuitive illustration of the signal-processing pipeline, Figure 3 presents representative examples linking raw audio input to its corresponding MFCC-based feature representation. Each example includes the input waveform, the derived MFCC (Mel) coefficients, and a qualitative interpretation of the observed acoustic patterns for both queen-present (QP) and queen-absent (QA) conditions. It should be noted that these examples are provided for illustrative purposes only and are intended to facilitate interpretation of the feature extraction process. They do not represent the full variability of the dataset.

### 3.3. Detection of Anomalies in Bee Hives Based on Human Audition and IA

**Figure 4:**
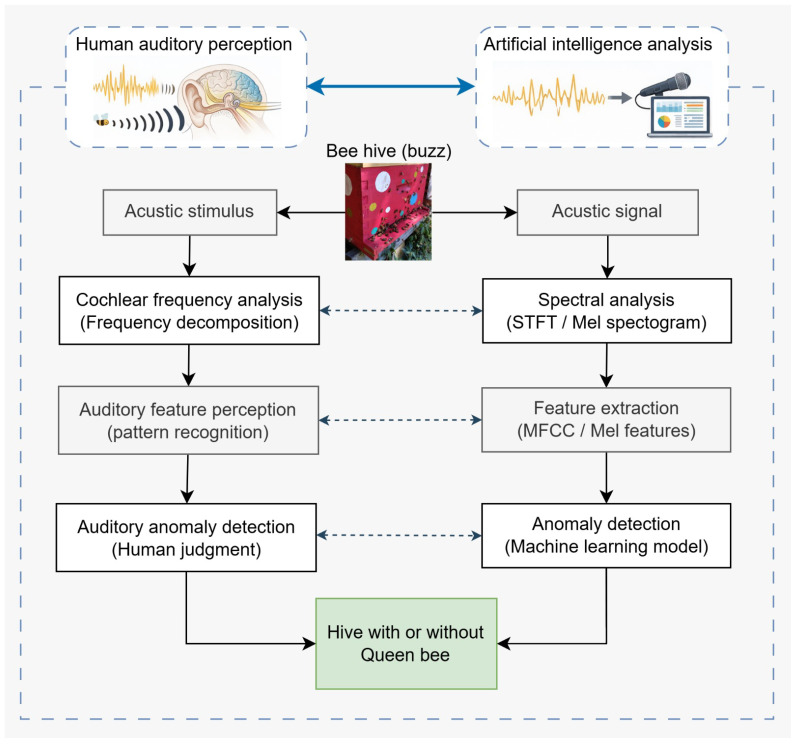
Conceptual analogy between human auditory perception and artificial intelligence models for acoustic-based anomaly detection in honeybee hives. Solid arrows indicate the primary processing flow within each domain (human perception on the left and artificial intelligence analysis on the right), while dashed arrows represent conceptual correspondences between analogous processing stages across the two domains.

A useful conceptual reference for designing artificial intelligence models to detect hive anomalies from sound is the human auditory system. In practice, experienced beekeepers can infer queen presence or absence by listening to hive buzzing patterns. Analogously, when sound waves reach the human ear, vibrations are processed in the cochlea, where different regions respond to different frequency bands, and perception follows a logarithmic rather than linear scale, as reflected by the Mel scale. Therefore, frequency-domain representations aligned with human perception are particularly suitable for acoustic analysis. Based on this rationale, Mel spectrograms and MFCCs are employed as feature extractors, while linear spectrograms are discarded. Finally, ML and DL models act as decision mechanisms, with feature extraction analogous to the beekeeper’s ear and the model inference to the human brain. This conceptual analogy is illustrated in Figure 4.

### 3.4. Preprocessing

#### 3.4.1. Feature Extraction

To implement the feature-extraction pipeline, the Python package librosa was used for audio processing, as it provides comprehensive tools for spectral analysis and perceptually motivated representations. In parallel, visual inspection of the extracted features was conducted using matplotlib, enabling systematic comparison across representations. For each sample, multiple descriptors were computed, including the amplitude time series, logarithmic spectrogram, Mel spectrogram, and MFCCs with 13, 20, and 40 coefficients. Moreover, MFCCs were derived both directly from the amplitude signal and from the Mel spectrogram. Consequently, this multi-representation analysis facilitated an informed assessment of which features most effectively capture discriminative acoustic patterns for accurate hive status classification.

The number of MFCCs was selected based on empirical and methodological considerations. First, numerous studies indicate that 13 MFCCs are sufficient to characterise human speech patterns, suggesting potential suitability for modelling bee colony buzzing. Second, prior work reported improved performance using 20 coefficients when analysing bee-related audio signals [44,45]. Together, these findings motivate the evaluation of both 13- and 20-coefficient configurations. Therefore, assessing whether increasing the number of coefficients to 40 improves or degrades classification performance relative to lower-dimensional representations becomes a relevant experimental question.

#### 3.4.2. Feature Selection

It is expected that not all extracted features contribute equally to classification performance. As a guiding principle, the human auditory system provides a useful reference, as trained listeners can effectively discriminate sound patterns. Accordingly, features that emulate auditory perception are preferable for audio-based models. According to [46], sound-induced vibrations are processed in the cochlea, where different regions respond to distinct frequency bands, and perception follows a logarithmic scale. Therefore, a frequency-domain approach aligned with perceptual scaling is advantageous. Based on this rationale, the amplitude time series was discarded, focusing on spectrogram-based representations. Although logarithmic spectrograms and Mel-based features both operate in the frequency domain, the latter better reflect human perception. Consequently, Mel spectrograms and MFCCs are expected to be most informative, while logarithmic spectrograms were retained for comparative validation.

### 3.5. Models

To evaluate the effectiveness of different learning paradigms for acoustic-based hive analysis, both classical ML and DL models were considered in this study. On the one hand, classical machine learning algorithms provide interpretable and computationally efficient baselines that have been successfully applied to pattern recognition tasks in bioacoustics and honeybee monitoring. On the other hand, deep learning models enable the automatic extraction of hierarchical representations from spectral features, offering greater modelling capacity for complex acoustic patterns. Accordingly, the selected models span probabilistic, margin-based, and neural network approaches, allowing a comprehensive comparison across different levels of model complexity. Table 2 summarises the classical machine learning and deep learning models evaluated in this study for acoustic-based honeybee hive classification, highlighting their underlying principles and application context [47].

### 3.6. Experimental Setup

Table 3 provides a concise overview of the experimental setup used for queen-presence classification.

#### Metrics

The performance of the proposed models was assessed using standard evaluation metrics for binary classification, as summarised in Table 4. The confusion matrix is a fundamental tool for evaluating binary classifiers, summarising prediction outcomes by comparing true and predicted labels. It comprises true positives (*TP*) and true negatives (*TN*), which represent correct predictions, and false positives (*FP*) and false negatives (*FN*), which indicate misclassifications.

### 3.7. Statistical Validation

Statistical validation was conducted using interval estimation to quantify the reliability and stability of the reported classification performance. Model performance is summarised using mean accuracy and standard deviation across evaluation runs, complemented by 95% confidence intervals computed via the Student’s t-distribution. These confidence intervals provide an estimate of the uncertainty associated with the mean accuracy and allow direct comparison of performance stability across acoustic features and segment lengths. By focusing on interval-based estimation rather than null-hypothesis significance testing, the analysis avoids assumptions about underlying performance distributions and emphasises the practical reliability of the observed results. This approach is particularly suitable for machine learning experiments under field conditions, where performance variability and limited sample sizes may challenge strict parametric assumptions. Although confidence intervals provide a robust estimation of performance variability, formal hypothesis testing (paired *t*-tests or non-parametric tests) was not conducted due to the limited number of evaluation repetitions. Consequently, the analysis focuses on interval-based interpretation rather than statistical significance testing. This choice prioritises robustness estimation under realistic experimental constraints while acknowledging that formal comparative testing remains an avenue for future work.

## 4. Results

This section reports experimental results for queen-presence classification using short-duration hive audio, evaluating classical and deep learning models across segment length, feature representation, and architecture. Beyond computational performance, the analysis is interpreted in the context of colony behavioural dynamics, as queen presence is closely associated with stabilised worker activity, pheromone-mediated regulation, and characteristic vibrational patterns within the hive. Performance trends in accuracy, robustness, and generalisation under realistic field conditions are summarised, followed by analyses of segment duration, feature type, and model choice, with statistical validation supporting the observed differences. Particular attention is given to the biological plausibility of the detected acoustic signatures and their relevance for non-invasive colony status assessment. Additional implementation details are provided in Appendix A.

### 4.1. Overview

Across all experiments, classification performance was strongly influenced by both segment length and acoustic feature representation. Short-duration segments (1 s) consistently achieved competitive or highest accuracy and agreement (0.726 accuracy and 0.345 Kappa, Table 5), supporting the feasibility of brief-sample characterisation of colony states, as also reported in prior bioacoustic studies [25]. Feature representations that preserve time–frequency structure, such as spectrograms and Mel-based variants, generally outperformed simpler coefficient-based descriptors by retaining richer harmonic and temporal information [24]. In terms of model architecture, convolutional neural networks consistently yielded the most robust performance across segment lengths, exhibiting improved generalisation under realistic field noise conditions and low-latency inference. Table 5 summarises the comparative performance of all evaluated configurations, highlighting the higher accuracy and agreement (0.726 vs. 0.250 accuracy; 0.345 vs. −0.435 Kappa) achieved by convolutional models in real-world hive monitoring scenarios.

**Figure 5:**
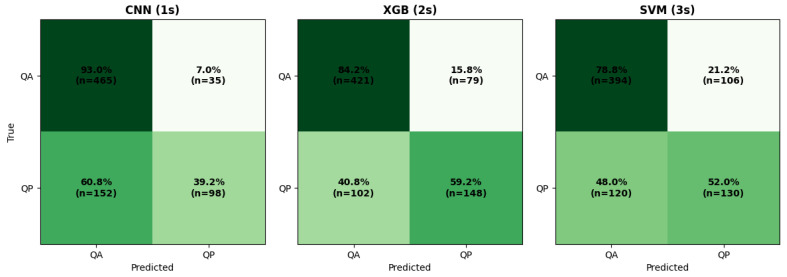
Normalised confusion matrices for MFCC (Mel)-based models across segment lengths (CNN–1 s, XGB–2 s, SVM–3 s). Values represent row-wise percentages, with absolute counts in parentheses. QA denotes queen absence and QP denotes queen presence.

To provide a more detailed interpretation of classification performance under class imbalance, Figure 5 presents the normalised confusion matrices for the best-performing MFCC (Mel)-based configurations across segment lengths. The results show that all models achieve strong discrimination for the majority class (QA), while performance for the minority class (QP) varies across models. In particular, the XGBoost model exhibits a more balanced distribution of true positives and true negatives, whereas the CNN model demonstrates higher specificity but lower sensitivity. These patterns highlight the importance of complementing accuracy with agreement-based metrics such as Cohen’s Kappa when evaluating model performance under imbalanced conditions.

**Figure 6:**
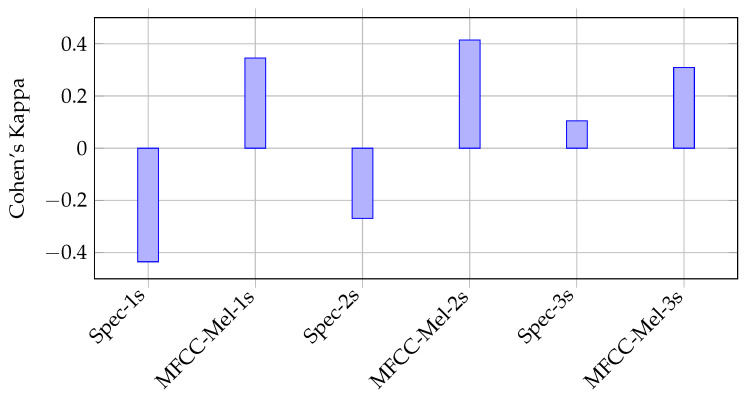
Cohen’s Kappa values for selected feature representations across segment lengths. Negative or near-zero Kappa values in spectrogram-based models indicate agreement at or below chance level, whereas MFCC (Mel) features consistently demonstrate stronger and more reliable agreement in queen-presence detection under field conditions.

Cohen’s Kappa was analysed to assess agreement beyond chance across feature representations and segment lengths. As shown in Figure 6, spectrogram-based configurations yielded negative or near-zero Kappa values for short segments, indicating poor agreement and unreliable discrimination under class imbalance. In contrast, MFCC (Mel) representations consistently achieved positive and substantially higher Kappa scores (0.345 vs. −0.435 and 0.414 vs. −0.269) across all segment durations. This pattern demonstrates that perceptually scaled cepstral features provide not only higher accuracy but also more reliable classification agreement, reinforcing their biological relevance for queen-presence detection under realistic field conditions.

### 4.2. Impact of Class Imbalance and Agreement Beyond Chance

As described before, the dataset exhibits class imbalance evaluation set (500 queen-absent vs. 250 queen-present samples), overall accuracy alone does not fully reflect discriminative performance. In several configurations, moderate accuracy values were accompanied by low or even negative Cohen’s Kappa scores, indicating agreement levels close to or below chance. This behaviour was particularly evident in spectrogram-based models under shorter segment lengths, where high misclassification rates of the minority class reduced true agreement. In contrast, MFCC (Mel) representations yielded consistently positive and higher Kappa values, suggesting improved balance between sensitivity and specificity. These results confirm that perceptually scaled features enhance agreement reliability in imbalanced acoustic classification scenarios. Under class imbalance, accuracy may overestimate performance by reflecting majority class dominance rather than true predictive ability. Metrics such as Cohen’s Kappa and recall provide more informative evaluation, as Kappa accounts for agreement beyond chance and recall captures the detection of minority class instances. Together, they offer a more balanced and reliable assessment of model performance under skewed distributions.

### 4.3. Effect of Segment Length (1 s vs. 2 s vs. 3 s)

Short-duration audio segments, particularly 1 s windows, achieved accuracy comparable to or exceeding longer segments. Importantly, this indicates that salient queen-related acoustic cues are embedded within brief temporal micro-patterns, rather than requiring extended signal aggregation. From a biological perspective, queen presence regulates worker behaviour through pheromone signalling, generating rapid vibrational and harmonic modulations within the colony soundscape. Accordingly, the superior performance of 1 s segments suggests that these short-lived spectral structures are effectively captured without temporal dilution. In addition, shorter windows reduce cumulative environmental noise and computational burden, supporting scalable real-time monitoring, especially for apiaries with a large number of hives to inspect. Taken together, these findings reflect a deliberate effort to identify the minimal biologically informative acoustic window capable of preserving discriminative power under realistic field conditions.

### 4.4. Perceptually Scaled Acoustic Features and Colony Behaviour

Mel-based representations consistently outperformed linear spectrograms, indicating that logarithmic frequency scaling more effectively captures biologically meaningful harmonic structures within hive acoustics. Honeybee colony soundscapes arise from overlapping vibrational components generated by wing beating, worker aggregation, and queen-associated behavioural regulation. Because these signals are distributed across frequency bands in a non-linear manner, perceptually scaled features provide a more faithful representation of their spectral organisation. Consequently, Mel-based descriptors enhance sensitivity to subtle modulation patterns linked to queen presence, while simultaneously reducing susceptibility to broadband environmental noise. Overall, these findings support the use of perceptually grounded acoustic representations for biologically interpretable hive-state classification.

### 4.5. Feature-Type Comparison and Analysis

Spectrograms were included as a baseline reference to evaluate the added value of perceptually scaled features. As a widely used time–frequency representation, they provide a standard point of comparison for assessing feature effectiveness. Their inclusion allows for a direct evaluation of whether perceptually motivated representations, such as Mel spectrograms and MFCCs, offer improved robustness and discriminative capacity, particularly under noisy and variable field conditions [38]. The comparative evaluation of acoustic representations revealed consistent performance differences across segment lengths and model architectures. Specifically, time–frequency descriptors incorporating perceptual scaling, particularly Mel-based features and MFCC Mel coefficients, achieved higher accuracy and agreement (0.726 vs. 0.250 accuracy; 0.345 vs. −0.435 Kappa) than linear spectrograms or simpler coefficient-based features. This suggests that logarithmic frequency mapping enhances the capture of harmonic and modulation patterns linked to queen presence, while reducing sensitivity to background noise and recording variability. In contrast, conventional spectrograms showed reduced robustness (accuracy 0.250 ± 0.039 with negative Kappa −0.435±0.109), especially for shorter segments, likely due to spectral sparsity and environmental interference. Moreover, variability analysis demonstrated that MFCC Mel features produced narrower confidence intervals (±0.015 vs. ±0.085, Table 6) and lower fold-to-fold variation. Taken together, these findings reflect our intention to identify biologically meaningful and statistically stable representations suitable for reliable hive monitoring in realistic field environments.

### 4.6. Model Comparison and Performance

The comparison of learning architectures revealed a consistent performance advantage for CNN across most feature representations and segment lengths. Specifically, CNN models achieved higher accuracy and agreement than classical machine learning approaches, demonstrating an enhanced ability to capture local time frequency structures and non linear interactions within hive acoustic signals. Although SVM and XGBoost showed competitive results for certain feature and segment combinations, their performance displayed greater variability under field noise. In contrast, CNN models maintained more stable agreement across conditions, suggesting improved resilience to recording heterogeneity. This robustness likely reflects the capacity of convolutional layers to model spatially organised spectral patterns associated with colony behavioural states. Importantly, stability under realistic recording environments strengthens the ecological validity of the proposed acoustic monitoring framework and supports its applicability to non invasive queen detection.

Model performance varied with segment length, reflecting differences in temporal information content and feature representation. For short segments (e.g., 1 s), convolutional neural networks (CNNs) showed stronger performance, likely due to their ability to capture localised time–frequency patterns and fine-grained acoustic structures. In contrast, for longer segments (2–3 s), classical machine learning models (e.g., SVM, XGBoost) achieved better results, as aggregated features such as MFCCs provide more stable statistical representations over extended time windows. This behaviour suggests that model effectiveness is inherently linked to temporal resolution. From a deployment perspective, these findings highlight the need to align model selection with the desired temporal granularity, or to consider hybrid or adaptive approaches that balance responsiveness and stability in real-world monitoring systems.

### 4.7. Precision–Recall Trade-Off Analysis

**Figure 7:**
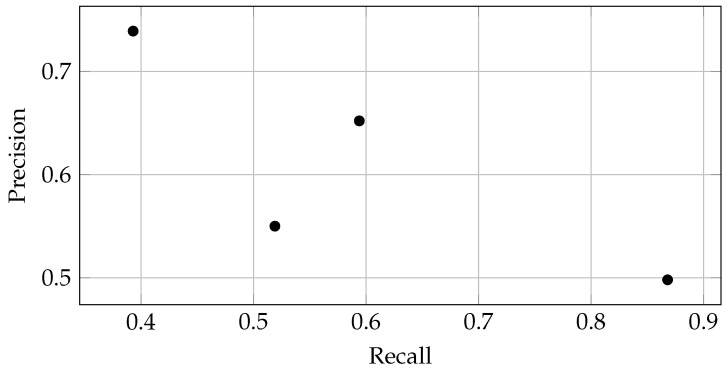
Precision–recall trade-off for the best-performing queen-presence classification models across segment lengths and feature representations. Each point represents a high-performing configuration, illustrating the balance between sensitivity to queen absence and precision in avoiding false alarms under realistic field conditions.

Analysis of precision and recall revealed distinct behavioural tendencies across models. Some configurations, particularly MFCC (Amplitude) with classical classifiers, achieved high recall but comparatively low precision, indicating aggressive detection of queen-present cases at the expense of false positives. In contrast, CNN-based models achieved a more balanced precision–recall profile (precision 0.739 with recall 0.393 vs. 0.498/0.868 imbalance), resulting in higher F1-scores and stronger agreement metrics. This balance is particularly important in queen-detection applications, where both missed detections and false alarms can negatively impact colony management decisions. Figure 7 presents the precision–recall distribution of the strongest configurations, demonstrating how different models balance sensitivity to queen-related acoustic cues with robustness against environmental noise.

A clear trade-off between precision and recall was observed across configurations. High recall, while desirable for detecting critical colony states, may lead to increased false positives, potentially triggering unnecessary and intrusive inspections. Conversely, lower recall reduces false alarms but increases the risk of missing relevant colony conditions. These results highlight the need to balance sensitivity and specificity according to the operational context, particularly in real-world beekeeping scenarios where both over-intervention and missed detections carry practical consequences.

### 4.8. Statistical Validation

Table 6 presents the classification accuracy of the best-performing model for each acoustic feature and segment length, including mean values, standard deviations, and 95% confidence intervals. Across all segment durations, MFCC-based representations consistently achieved higher accuracy (0.726 vs. 0.608 vs. 0.250) and narrower confidence intervals than spectrogram-based features, indicating superior performance and greater stability. The highest accuracy was obtained using MFCC (Mel) features with convolutional neural networks for 1 s segments and gradient-boosted trees for 2 s segments, while support vector machines performed best for 3 s segments. In contrast, spectrogram-based models showed lower accuracy and wider confidence intervals, particularly for shorter segments, reflecting increased variability under field conditions. Overall, the confidence intervals provide a robust measure of estimation reliability and support the selection of MFCC (Mel) features for queen-presence detection across different temporal resolutions.

### 4.9. Implications for Beekeeping Practice

From a practical perspective, reliable detection of queen absence within short acoustic windows enables rapid colony assessment without invasive inspection. While false positives may prompt unnecessary hive inspection, false negatives could delay corrective action following queen loss. Therefore, configurations achieving balanced precision and recall are particularly valuable for sustainable colony management.

## 5. Discussion

This study demonstrates that queen presence can be reliably detected from short-duration acoustic segments (1–3 s) under realistic field conditions. The findings demonstrate that queen presence can be reliably inferred from short-duration hive audio using perceptually grounded acoustic representations and appropriate classification models. Beyond computational performance, these results provide insight into how colony-level behavioural dynamics are reflected in the acoustic structure of the hive soundscape. Because queen pheromone signalling plays a central role in regulating worker organisation, brood care, and colony stability, detectable changes in vibrational and harmonic patterns are expected following queen loss. The observed superiority of specific feature representations therefore supports the biological plausibility of acoustic queen monitoring and highlights its potential as a non-invasive tool for improving colony management under realistic field conditions. Accordingly, the reported performance differences should be interpreted as indicative rather than statistically conclusive. In line with these observations, the illustrative examples in Figure 3 provide a qualitative complement by highlighting differences in spectral structure between QP and QA conditions.

### 5.1. Interpretation of Findings

The results indicate that both acoustic feature representation and temporal resolution significantly influence model performance. Across segment lengths, MFCC-based configurations consistently achieved higher accuracy and narrower confidence intervals than spectrograms, suggesting that perceptually motivated features effectively capture queen-related cues while reducing sensitivity to noise. Notably, no single algorithm dominated; CNNs excelled with 1 s segments, gradient-boosted trees with 2 s, and SVMs with 3 s, emphasizing that model suitability depends on temporal granularity. In contrast, spectrograms were less robust, particularly with shorter segments. Overall, these findings support the feasibility of real-time, non-invasive queen detection through carefully chosen features and models.

Queen presence within a colony is associated with stabilised worker behaviour, reduced agitation signals, and characteristic vibrational harmonics modulated by pheromonal communication. In contrast, queen loss often induces increased worker excitation, irregular buzzing intensity, and altered frequency dispersion patterns. The superior performance of MFCC (Mel) representations suggests that cepstral features effectively capture these harmonic density shifts and modulation patterns rather than isolated frequency peaks. This supports the hypothesis that queen-related acoustic differences manifest as global spectral texture changes rather than discrete tonal events.

From a beekeeping perspective, the balance between sensitivity and precision is critical. False negatives (undetected queen absence) may delay intervention and increase colony stress or collapse risk, whereas false positives may trigger unnecessary hive inspections. Configurations achieving balanced precision–recall profiles are therefore preferable to those optimising accuracy alone. The stronger agreement metrics observed in MFCC (Mel)-based models indicate improved reliability for practical queen-monitoring applications.

Hive vibroacoustic signals are inherently short-duration events, supporting the use of 1–3 s analysis windows. Prior studies report that queen signals (tooting and quacking) and worker-produced signals (piping, stop signals) typically last between 0.1 and 1 s and are fully captured within this temporal range [48]. Moreover, worker piping—more frequent in queenless colonies—occurs in rapid, repeated emissions, allowing multiple complete signals to be recorded within a 3 s window. Consequently, short windows preserve biologically meaningful temporal and spectral patterns, providing sufficient information to characterise colony state.

### 5.2. Comparison with Prior Work

Compared with prior bioacoustic studies, which typically rely on long-duration recordings (30–90 s) or data collected under controlled laboratory conditions [18], the present results demonstrate that short audio segments (1–3 s) are sufficient for accurate queen-presence detection in realistic field environments [45]. While earlier research has primarily addressed general hive status monitoring or swarm activity detection, relatively few studies have explicitly focused on queen absence using perceptually grounded acoustic features [49]. The strong performance of MFCC (Mel)-based representations, particularly when combined with convolutional neural networks and gradient-boosted models, is consistent with previous findings showing that Mel-scaled features better capture biologically relevant frequency patterns and exhibit increased robustness to environmental noise [50]. In addition, the use of audio data aggregated from multiple independent sources (Hiveeyes, LongHive, and the USM Bee Lab) extends prior single-dataset approaches and provides evidence that cross-site generalisation can be achieved through consistent preprocessing and feature extraction strategies.

Unlike prior studies based on extended recording windows [18], the present findings indicate that queen-related acoustic information is embedded within short temporal micro-patterns. Consequently, brief segments can support continuous field monitoring while reducing storage demands and enabling rapid anomaly detection without compromising biological interpretability. Importantly, early detection of queen loss strengthens colony resilience and stabilises brood dynamics, which are critical for pollination services and agricultural productivity [51]. In this context, our work aims to contribute to scalable, non-invasive monitoring strategies capable of supporting sustainable apiary management under growing environmental pressures.

### 5.3. Applications and Deployment

The findings support the feasibility of real-time, low-cost hive monitoring for early queen detection. Specifically, the effectiveness of short audio segments, particularly when combined with MFCC representations, enables deployment on embedded and low-power platforms with minimal computational demand. Consequently, integration into wireless sensor networks can provide continuous acoustic surveillance and timely alerts following queen loss. Importantly, early detection helps preserve colony productivity, brood stability, and pollination efficiency while reducing the need for intrusive inspections. In this regard, the proposed framework was designed to contribute to scalable, non-invasive monitoring strategies adaptable to diverse apiary environments.

The proposed approach achieves accuracy values up to approximately 0.73, demonstrating its feasibility for practical, low-cost hive monitoring under realistic field conditions. While performance varies across configurations, the results highlight a clear trade-off between sensitivity and specificity, particularly under class imbalance and environmental noise. These findings suggest that the system can support early-stage decision-making in beekeeping contexts, where moderate accuracy combined with robust behaviour under heterogeneous conditions is often preferable to highly optimised but less generalizable solutions.

### 5.4. Limitations

Despite promising results, several limitations warrant consideration. First, dataset size and hive diversity were limited, potentially restricting generalisation across climates, apiary configurations, and subspecies. Second, environmental noise and recording hardware variability may have influenced feature quality under field conditions. Moreover, the limited number of evaluation repetitions reduces statistical power, justifying the use of confidence intervals rather than formal hypothesis testing. In addition, the current binary framework does not capture broader colony events such as swarming or predator activity. Importantly, the absence of direct behavioural or pheromone measurements limits causal interpretation between acoustic patterns and colony physiology, an aspect we aim to address in future work. Model performance may vary across bee subspecies and climatic conditions, highlighting the need for large-scale cross-regional validation.

A key limitation of this study is the absence of an explicit decomposition of the impact of individual urban noise sources on classification performance. Although recordings were acquired under moderate urban environmental noise conditions (including traffic, wind, and human activity), the dataset does not include controlled annotations or isolated measurements of these sources. As a result, the influence of specific noise types cannot be independently quantified. While perceptually scaled representations (MFCCs derived from Mel spectrograms) demonstrated robustness under heterogeneous acoustic conditions, this robustness is inferred from aggregate performance rather than causal analysis [18]. Future work should incorporate controlled noise injection or stratified evaluation protocols to systematically assess the effect of distinct environmental noise factors and their intensity on model performance.

Differences in recording devices and compression formats may introduce variability that influences feature extraction and model performance. Such heterogeneity, including microphone characteristics and encoding artifacts, represents a real-world robustness challenge, as models may partially capture device-specific patterns rather than purely biological signals.

In addition, the absence of formal statistical significance testing limits the ability to establish definitive performance differences between models.

### 5.5. Future Work

Future research should prioritise expanding acoustic datasets across diverse climates, seasons, apiary contexts, and honeybee subspecies to improve generalisation. Furthermore, semi-supervised and self-supervised approaches may leverage large volumes of unlabeled hive audio to enhance representation learning with reduced annotation effort. In addition, multi-task frameworks could jointly detect queen absence, swarming, predator intrusion, and stress-related states. Methodologically, increasing evaluation repetitions would enable stronger statistical comparison through paired testing and effect-size analysis. Finally, lightweight architectures and optimised feature pipelines should be explored for on-device inference, together with interpretable visual tools to facilitate practical adoption by beekeepers.

## 6. Conclusions

This study demonstrates that short-duration acoustic analysis constitutes a viable and non-invasive approach for monitoring queen presence in honeybee colonies. By integrating multisource audio datasets (Hiveeyes, LongHive, and the USM Bee Lab) and systematically evaluating classical machine learning and deep learning models, the results show that reliable queen-presence detection can be achieved under realistic field conditions using brief audio segments.

The experimental findings indicate that acoustic feature representation plays a more critical role than model complexity. In particular, MFCC-based representations—especially MFCC (Mel)—consistently outperformed spectrogram-based features across segment lengths, achieving higher accuracy and narrower confidence intervals. Convolutional neural networks yielded the best performance for 1 s segments, while gradient-boosted models and support vector machines performed optimally at longer temporal resolutions, highlighting that model effectiveness is closely tied to segment duration. These results confirm that one-second audio windows contain sufficient information for accurate classification, enabling low-latency processing suitable for real-time monitoring.

Overall, the proposed framework provides a scalable foundation for intelligent hive monitoring systems and supports the use of perceptually motivated acoustic features for robust deployment in noisy, real-world environments. Future work should extend data collection across broader climatic and seasonal contexts, investigate multi-event detection scenarios, and explore lightweight model architectures for embedded deployment. Integrating AI-based acoustic diagnostics into beekeeping practices has the potential to enhance early anomaly detection and contribute to the sustainability and resilience of apiculture.

## Figures and Tables

**Table 1:** Summary of frequency-domain and perceptually motivated audio representations employed for acoustic feature extraction.

Method	Description
Discrete Fourier Transform (DFT)	A mathematical transformation that converts a time-domain signal into its frequency-domain representation [40]. In this study, it is used to analyse colony buzz recordings by expressing signal amplitude as a function of frequency.
Fast Fourier Transform (FFT)	A family of efficient algorithms for computing the DFT with computational complexity O(nlogn), most commonly implemented using the Cooley–Tukey algorithm [41]. Widely applied in audio and image signal processing.
Spectrogram	A time–frequency representation obtained by applying the DFT (typically via FFT) over successive signal windows. Time is shown on the X-axis, frequency on the Y-axis, and signal energy is represented by colour intensity, supporting feature extraction and acoustic analysis.
Mel Scale	A perceptual frequency scale reflecting human auditory sensitivity, where frequencies below 1000 Hz are perceived linearly and higher frequencies logarithmically, emphasizing discrimination at lower frequencies.
Mel Spectrogram	A spectrogram whose frequency axis is transformed using the Mel scale, providing a perceptually motivated representation of sound. Time is shown on the X-axis, Mel-scaled frequency on the Y-axis, and intensity is measured in decibels.
Mel-Frequency Cepstral Coefficients (MFCCs)	Compact feature representations derived from Mel-scaled spectra, widely used in speech and bioacoustic analysis due to their alignment with human auditory perception.

**Table 2:** Machine learning and deep learning models evaluated for hive acoustic classification.

Category	Model	Description
ML	Extreme Gradient Boosting (XGBoost, XGB)	Ensemble learning method based on gradient-boosted decision trees, optimised through regularisation and tree pruning to improve predictive performance and generalisation; widely applied in structured data classification and bioacoustic monitoring tasks.
ML	Support Vector Machine (SVM)	Margin-based classifier effective in high-dimensional feature spaces; commonly employed for acoustic and bioacoustic classification problems.
DL	Convolutional Neural Network (CNN)	Neural architecture capable of learning hierarchical spatial patterns from time–frequency representations such as spectrograms; well suited for acoustic analysis in hive monitoring.
DL	Multilayer Perceptron (MLP)	Fully connected neural network that models non-linear relationships between extracted features; used as a baseline deep learning approach for classification tasks.

**Table 3:** Summary of the experimental setup for queen-presence classification.

Step	Description
Objective	Build, train, and evaluate convolutional models for queen-presence classification using short hive-audio segments with MFCC and spectrogram-based features under realistic noise conditions.
Data Sources	Multi-source audio from Hiveeyes (Berlin, 11 hives, 20 recordings, .mp3, 44,100 Hz), LongHive, and USM Bee Lab (Valparaíso). Classes: CA (queen present), SA (queen absent).
Labeling	Files renamed as #-status (# unique ID; status ∈{CA,SA}). Label balance verified per source and segment length.
Segmentation	Recordings split into 1, 2, and 3 s windows (non-overlapping). Datasets created per duration and divided into 80% training and 20% testing sets.
Feature Extraction	Using librosa: (1) Logarithmic spectrograms, (2) Mel spectrograms, (3) MFCCs from waveform (13, 20, 40), (4) MFCCs from Mel spectrogram (13, 20, 40). Visualised with matplotlib.
Feature Selection	Focused on perceptual features (Mel, MFCCs) consistent with human hearing; log-spectrogram included as baseline control.
Modeling Approach	Adopted pre-existing CNN architecture (by mikesmales) for MFCC matrices. Transfer learning considered but unnecessary after direct training on generated datasets.
CNN Architectures	Model A: native for 40 MFCCs (trainable with 20). Model B: adapted version supporting 13/20/40 MFCCs by removing final Conv/Pooling/Dropout layers. Both tested on log- and Mel-spectrograms.
Hyperparameters	Activations: ReLU (hidden), Softmax (output); Loss: categorical cross-entropy; Optimiser: Adam; Epochs: 72; Batch size: 32; Dropout: 0.2. Dense layers—Model A: 80–2080–8256–32896–258; Model B: 80–2080–8256–130.
Training Protocol	Separate runs per feature type (MFCC 13/20/40, log-, Mel-spectrogram) and segment duration (1/2/3 s). Training on Google Colab GPU. Accuracy used as main metric.
Evaluation	Metrics: accuracy, precision, recall, specificity (Eqs. 1–4). Confusion matrices generated for best models.
Reproducibility	Implemented in Python (v3.10) using librosa (v0.10), numpy (v1.24), matplotlib (v3.7), and deep learning frameworks such as TensorFlow/Keras (v2.12) or PyTorch (v2.0). Random seeds were fixed, and configurations were logged for each run.
Artifacts	Trained weights, logs, and evaluation outputs stored. Best model and preprocessing pipeline exported for deployment.

**Table 4:** Evaluation metrics for binary classification (queen present vs. absent).

Metric	Formula	Description
Accuracy	$\frac{TP+TN}{TP+TN+FP+FN}$	Proportion of correctly classified instances among all samples.
Precision	$\frac{TP}{TP+FP}$	Fraction of predicted positives that are truly positive (model reliability).
Recall	$\frac{TP}{TP+FN}$	Ability of the model to identify all positive (queen-present) cases.
F1-score	$\frac{2TP}{2TP+FP+FN}$	Harmonic mean of precision and recall, balancing false positives and false negatives in queen-presence classification.
Cohen’s Kappa	$\frac{P_{o}-P_{e}}{1-P_{e}}$	Agreement between predicted and true labels corrected for chance, derived from the confusion matrix marginals.

**Table 5:** Best-performing configuration per acoustic feature and segment length. Results are reported as mean ± standard deviation. (760 test sample (500 negative–250 positive)).

Segment	Feature	Model	Accuracy	Precision	Recall	F1-Score	Kappa
1 s	Spectrogram	SVM	0.250±0.039	0.171±0.066	0.280±0.122	0.213±0.085	−0.435±0.109
	Mel-spectrogram	XGB	0.608±0.022	0.460±0.036	0.343±0.034	0.391±0.009	0.112±0.026
	MFCC (Amplitude)	SVM	0.630±0.004	0.498±0.005	0.868±0.043	0.633±0.015	0.311±0.014
	MFCC (Mel)	CNN	0.726±0.015	0.739±0.015	0.393±0.064	0.511±0.053	0.345±0.047
2 s	Spectrogram	XGB	0.417±0.147	0.214±0.163	0.150±0.037	0.169±0.077	−0.269±0.251
	Mel-spectrogram	XGB	0.591±0.003	0.418±0.023	0.263±0.025	0.322±0.023	0.051±0.018
	MFCC (Amplitude)	SVM	0.637±0.018	0.506±0.017	0.887±0.038	0.644±0.020	0.326±0.033
	MFCC (Mel)	XGB	0.732±0.041	0.652±0.046	0.594±0.079	0.621±0.060	0.414±0.089
3 s	Spectrogram	SVM	0.654±0.085	0.547±0.233	0.203±0.025	0.287±0.057	0.105±0.150
	Mel-spectrogram	XGB	0.640±0.006	0.453±0.016	0.357±0.049	0.398±0.033	0.146±0.023
	MFCC (Amplitude)	NB	0.582±0.008	0.439±0.008	0.893±0.014	0.589±0.010	0.253±0.014
	MFCC (Mel)	SVM	0.697±0.008	0.550±0.008	0.519±0.042	0.533±0.026	0.309±0.028

**Table 6:** Accuracy performance with 95% confidence intervals for the best-performing configuration per acoustic feature and segment length. Results are reported as mean ± standard deviation.

Segment	Feature	Model	Accuracy (95% CI)
1 s	Spectrogram	SVM	0.250±0.039 [0.202–0.298]
	Mel-spectrogram	XGB	0.608±0.022 [0.581–0.635]
	MFCC (Amplitude)	SVM	0.630±0.004 [0.625–0.635]
	MFCC (Mel)	CNN	0.726±0.015 [0.707–0.745]
2 s	Spectrogram	XGB	0.417±0.147 [0.234–0.600]
	Mel-spectrogram	XGB	0.591±0.003 [0.587–0.595]
	MFCC (Amplitude)	SVM	0.637±0.018 [0.615–0.659]
	MFCC (Mel)	XGB	0.732±0.041 [0.681–0.783]
3 s	Spectrogram	SVM	0.654±0.085 [0.548–0.760]
	Mel-spectrogram	XGB	0.640±0.006 [0.633–0.647]
	MFCC (Amplitude)	NB	0.582±0.008 [0.572–0.592]
	MFCC (Mel)	SVM	0.697±0.008 [0.687–0.707]

## Data Availability

All datasets and preprocessing scripts supporting the conclusions of this article will be made publicly available in an open-access repository upon acceptance, ensuring reproducibility of the acoustic feature extraction and classification procedures.

## Abbreviations

The following abbreviations are used in this manuscript:

AI	Artificial Intelligence
CNN	Convolutional Neural Network
DL	Deep Learning
MFCC	Mel-Frequency Cepstral Coefficients
XGB	Extreme Gradient Boosting
SVM	Support Vector Machine
MLP	Multilayer Perceptron

## Appendix A. Additional Implementation Details

This appendix reports the complete set of experimental results obtained in this study, covering all evaluated combinations of acoustic feature representations, segment lengths (1 s, 2 s, and 3 s), and machine learning and deep learning models. Table A1 provides detailed performance metrics, including accuracy, precision, recall, F1-score, and Cohen’s Kappa, expressed as mean and standard deviation across validation runs. The purpose of this appendix is to ensure full transparency and reproducibility of the experimental analysis, allowing readers to inspect the behaviour of individual models beyond the best-performing configurations discussed in the main text. In particular, the table highlights the impact of feature choice and temporal resolution on classification performance, as well as the variability and agreement patterns observed across different modelling approaches. For clarity and readability, only representative best-performing configurations per feature and segment length are summarised in the Section 4 while the present appendix preserves the complete evaluation landscape.

**Table A1:** Best metrics results.

			Accuracy	Precision	Recall	F1-Score	Kappa
1 [s]	SPECTROGRAM	SVM	0.250±0.039	0.171±0.066	0.280±0.122	0.213±0.085	−0.435±0.109
		XGB	0.249±0.035	0.119±0.005	0.163±0.012	0.137±0.004	−0.497±0.035
		MLP	0.206±0.016	0.151±0.051	0.259±0.106	0.191±0.070	−0.497±0.074
		CNN	0.159±0.012	0.040±0.007	0.056±0.010	0.047±0.008	−0.669±0.023
	MEL SPECTROGRAM	SVM	0.495±0.050	0.281±0.024	0.233±0.043	0.252±0.013	−0.121±0.057
		XGB	0.608±0.022	0.460±0.036	0.343±0.034	0.391±0.009	0.112±0.026
		MLP	0.473±0.108	0.295±0.070	0.274±0.026	0.279±0.024	−0.130±0.146
		CNN	0.509±0.075	0.302±0.065	0.237±0.046	0.262±0.039	−0.097±0.109
	MFCC AMPLITUDE	SVM	0.630±0.004	0.498±0.005	0.868±0.043	0.633±0.015	0.311±0.014
		XGB	0.380±0.002	0.250±0.022	0.346±0.052	0.290±0.033	−0.235±0.023
		MLP	0.403±0.027	0.335±0.015	0.634±0.037	0.438±0.017	−0.082±0.030
		CNN	0.314±0.045	0.118±0.091	0.143±0.121	0.130±0.104	−0.432±0.128
	MFCC MEL SPEC	SVM	0.565±0.003	0.088±0.018	0.019±0.003	0.032±0.006	−0.117±0.013
		XGB	0.719±0.015	0.623±0.038	0.609±0.038	0.614±0.006	0.395±0.020
		MLP	0.600±0.074	0.475±0.097	0.512±0.056	0.487±0.043	0.163±0.115
		CNN	0.726±0.015	0.739±0.015	0.393±0.064	0.511±0.053	0.345±0.047
2 [s]	SPECTROGRAM	SVM	0.235±0.027	0.179±0.024	0.296±0.043	0.223±0.031	−0.445±0.052
		XGB	0.417±0.147	0.214±0.163	0.150±0.037	0.169±0.077	−0.269±0.251
		MLP	0.187±0.009	0.147±0.024	0.252±0.062	0.186±0.036	−0.524±0.051
		CNN	0.224±0.133	0.160±0.170	0.332±0.411	0.214±0.242	−0.458±0.352
	MEL SPECTROGRAM	SVM	0.475±0.020	0.300±0.017	0.314±0.025	0.307±0.021	−0.116±0.039
		XGB	0.591±0.003	0.418±0.023	0.263±0.025	0.322±0.023	0.051±0.018
		MLP	0.541±0.049	0.335±0.072	0.233±0.036	0.275±0.049	−0.046±0.092
		CNN	0.483±0.094	0.254±0.085	0.172±0.030	0.198±0.012	−0.165±0.138
	MFCC AMPLITUDE	SVM	0.637±0.018	0.506±0.017	0.887±0.038	0.644±0.020	0.326±0.033
		XGB	0.385±0.054	0.180±0.085	0.188±0.102	0.183±0.092	−0.309±0.126
		MLP	0.360±0.044	0.236±0.110	0.373±0.235	0.287±0.154	−0.258±0.173
		CNN	0.313±0.035	0.055±0.042	0.052±0.037	0.054±0.039	−0.484±0.064
	MFCC MEL SPEC	SVM	0.549±0.036	0.178±0.073	0.056±0.022	0.086±0.033	−0.120±0.063
		XBG	0.732±0.041	0.652±0.046	0.594±0.079	0.621±0.060	0.414±0.089
		MLP	0.546±0.031	0.405±0.033	0.486±0.075	0.441±0.047	0.063±0.069
		CNN	0.577±0.152	0.533±0.280	0.474±0.087	0.464±0.057	0.127±0.229
3 [s]	SPECTROGRAM	SVM	0.232±0.015	0.139±0.040	0.252±0.079	0.179±0.054	−0.440±0.062
		XGB	0.654±0.085	0.547±0.233	0.203±0.025	0.287±0.057	0.105±0.150
		MLP	0.265±0.072	0.216±0.105	0.499±0.286	0.301±0.156	−0.284±0.220
		CNN	0.167±0.023	0.051±0.040	0.087±0.071	0.065±0.051	−0.607±0.064
	MEL SPECTROGRAM	SVM	0.423±0.049	0.226±0.011	0.294±0.031	0.254±0.006	−0.181±0.077
		XGB	0.640±0.006	0.453±0.016	0.357±0.049	0.398±0.033	0.146±0.023
		MLP	0.393±0.069	0.237±0.033	0.372±0.134	0.286±0.057	−0.196±0.060
		CNN	0.616±0.026	0.369±0.143	0.330±0.223	0.340±0.195	0.085±0.158
	MFCC AMPLITUDE	SVM	0.582±0.008	0.439±0.008	0.893±0.014	0.589±0.010	0.253±0.014
		XBG	0.384±0.057	0.084±0.068	0.102±0.106	0.091±0.084	−0.370±0.039
		MLP	0.366±0.036	0.181±0.053	0.262±0.112	0.213±0.073	−0.291±0.078
		CNN	0.314±0.028	0.029±0.030	0.033±0.033	0.031±0.031	−0.497±0.047
	MFCC MEL SPEC	SVM	0.563±0.030	0.255±0.160	0.208±0.153	0.228±0.159	−0.055±0.127
		XGB	0.697±0.008	0.550±0.008	0.519±0.042	0.533±0.026	0.309±0.028
		MLP	0.598±0.060	0.371±0.129	0.335±0.168	0.350±0.151	0.063±0.177
		CNN	0.617±0.131	0.517±0.266	0.394±0.073	0.417±0.063	0.140±0.198
